# Configural frequency analysis as a method of determining patients' preferred decision-making roles in dialysis

**DOI:** 10.1186/1472-6947-10-47

**Published:** 2010-09-11

**Authors:** Sabine Loeffert, Oliver Ommen, Christine Kuch, Fueloep Scheibler, Andrej Woehrmann, Conrad Baldamus, Holger Pfaff

**Affiliations:** 1 Center for Health Services Research Cologne, University of Cologne, Germany, Eupener Str. 129, 50933 Cologne, Germany; 2 Institute for Medical Sociology, Health Services Research and Rehabilitation Science (IMVR), University of Cologne, Eupener Str. 129, 50933 Cologne, Germany; 3 Institute for Quality and Efficiency in Health Care, Cologne, Germany; 4 Kuratorium fuer Dialyse und Nierentransplantation (KFH), Neu-Isenburg, Germany; 5 Department of Internal Medicine IV (retired), University Hospital of Cologne, Germany

## Abstract

**Background:**

Numerous studies examined factors in promoting a patient preference for active participation in treatment decision making with only modest success. The purpose of this study was to identify types of patients wishing to participate in treatment decisions as well as those wishing to play a completely active or passive role based on a Germany-wide survey of dialysis patients; using a prediction typal analysis method that defines types as configurations of categories belonging to different attributes and takes particularly higher order interactions between variables into account.

**Methods:**

After randomly splitting the original patient sample into two halves, an exploratory prediction configural frequency analysis (CFA) was performed on one-half of the sample (n = 1969) and the identified types were considered as hypotheses for an inferential prediction CFA for the second half (n = 1914). 144 possible prediction types were tested by using five predictor variables and control preferences as criterion. An α-adjustment (0.05) for multiple testing was performed by the Holm procedure.

**Results:**

21 possible prediction types were identified as hypotheses in the exploratory prediction CFA; four patient types were confirmed in the confirmatory prediction CFA: patients preferring a passive role show low information seeking preference, above average trust in their physician, perceive their physician's participatory decision-making (PDM)-style positive, have a lower educational level, and are 56-75 years old (Type 1; *p *< 0.001) or > 76 years old (Type 2; *p *< 0.001). Patients preferring an active role show high information seeking preference, a higher educational level, and are < 55 years old. They have either below average trust, perceive the PDM-style negative (Type 3; *p *< 0.001) or above average trust and perceive the PDM-style positive (Type 4; *p *< 0.001).

**Conclusions:**

The method prediction configural frequency analysis was newly introduced to the research field of patient participation and could demonstrate how a particular control preference role is determined by an association of five variables.

## Background

### Patients' preferences for involvement in treatment decisions

According to Charles and colleagues [1], there are three different models for describing patient-physician interaction. In the "paternalistic model", the physician makes treatment decisions to the best of his knowledge for the good of the patient without including the patient in the decision-making process. In the meantime, the patient passively acquiesces to the professional authority of the physician by agreeing to the physician's choice of treatment. In the "informed model", the physician is responsible for providing patients with relevant information on all treatment options as well as their benefits and risks, while the patients are then responsible for making the treatment decision. Unlike in the two previous models, in the "shared model", both the physician and the patient have an equal share in the decision-making process. As such, there is a two-way exchange of information between physician and patient and both agree on the final treatment decision [2].

The need to establish a physician-patient partnership when making treatment decisions is less compelling in the case of everyday problems with clear diagnosis and treatment paths than in the case of chronic, serious or life-threatening illnesses, for which there are several treatment options with different risks and benefits [1]. Shared decision making (SDM) has already shown to lead to improved treatment outcomes for chronically ill patients [3]. One-time treatment decisions made for the treatment of short, acute illnesses are often completely different from recurrent treatment decisions made for the therapy of chronic illnesses [4]. Alongside the well-known factors of physician-patient communication and of patient contributions and efforts, recent studies on the involvement of patients in making medical decisions have identified a third key factor: the contributions and efforts of the physicians involved [4,5]. The addition of the physicians' emphasis to the process of establishing a partnership is seen as a critical point in adapting SDM to chronic care decision making. During treatment of chronically ill patients, physicians and patients can engage in an ongoing information exchange followed by deliberation followed by more information exchange followed by further deliberation and so forth, while trust and partnership evolve alongside [4]. Other researchers suggest that physicians vary widely in how much they facilitate patients' active participation in treatment decisions, what they have termed a "participatory decision-making style" [6].

Shared decision making has gained importance as an approach to physician-patient communication and patient participation in recent years. For this reason, several self-report and objective scales for assessing aspects of patient participation already exist [7]. From a patient's perspective, the control preference construct measures "the degree of control an individual wants to assume when decisions are made about medical treatment" [8]. In the process patients are presented five descriptions of different roles in treatment decision making, which they have to consider in expressing their preferences.

Numerous studies have already examined the question as to whether and to what extent patients wish to take part in making decisions about their own health [9-15]. However, there are only few studies that report the decision making role preferences of dialysis patients. In this case, 42% of both Canadian and English participants in a study showed a definite desire to participate in treatment decision making, while 35% of Canadian patients and only 19% of English patients preferred to play an active role [16,17]. Two large US studies using a slightly different measurement method revealed that more than half of the participating patients (54%, 76% respectively) wanted to have at least a part of the responsibility in decision making [18,19]. Initial studies of the state and developmental tendencies of SDM among patients with end-stage renal disease (ESRD) in Germany showed that dialysis patients were greatly willing to play an active role in the decision-making process [20].

Numerous studies, which have examined the desired involvement of patients in making treatment decisions, have used univariate or multivariate analyses in an attempt to identify determinants of the preference for active participation in treatment decision making. The results of these studies are not quite clear, yet they indicate several tendencies. For instance, various studies were able to confirm that the older the patients were, the less likely they were to prefer an active role [9,10,21-24]. In contrast, yet other studies demonstrated that the age of patients had no influence on the patients' preferred role [12,25]. Studies also showed that a higher level of education attained by patients is positively associated with preferences for active participation [9,12,15,22,24-26] or has no influence [10] on the patients' desire to participate. In cases of newly established physician-patient relationships, the involvement of patients in making treatment decisions significantly increased the patients' level of trust in their physicians [27], while patients that had already developed a high level of trust expressed less of a desire to play an active role in decision making [28,29]. When asked how their medical treatment could be improved, patients responded that their top priority is to get more information from their treating physician [30]. This does not mean, however, that patients with a heightened need for information also necessarily wish to take on responsibility for their treatment decisions [22,30,31].

### Configural frequency analysis

Configural frequency analysis (CFA) is a procedure for analyzing multivariate cross-classifications that can be used in a wide range of multivariate experimental designs. Among the advantages of CFA often mentioned are: it is suitable for data on a nominal scale level and it is distribution-free. It allows further a profile-oriented analysis, which means the unit of an analysis is the profile or configuration of all observed values of a person [32]. In contrast to latent class analysis, CFA operates at the level of manifest variables and latent classes are neither assumed nor created. Further, CFA aims at identification of outstanding cells of multivariate cross-classifications instead of fitting a model. It seeks to identify patterns that stand out as more frequent (CFA types) or less frequent (CFA antitypes) than expected by chance [33]. Another advantage is that unlike many other methods, CFA does not use a similarity or distance measure to identify types, but takes only types into consideration with identical attribute patterns, which makes its type definition more accurate than any other previous type definition [34]. Further, other methods, like factor-analytical methods, yield a clustering of variables and not of persons. Another problem with typal analyses based on correlation matrices (like factor analysis) is that they take into consideration only first-order interactions of the variables. All types that are caused by higher-order interactions alone cannot be detected by these methods [34]. Configural frequency analysis has already been widely used in psychological, social, and medical research: CFA has proven to be useful in determining Wechsler Profile Types [35] and classifying headache syndromes [36,37]. Other researchers succeeded in predicting constellations of social support at work in the development of low back pain by CFA [38] or finding factors associated with the transition from abuse to dependence among substance abusers [39]. Further, CFA was used for examining adult psychopathy and violent behaviour in males with early neglect and abuse [40] or exploring bacterial sets in periodontal health and disease [41]. Other studies used CFA for examining histologic criteria and tumour behaviour of patients with gastrointestinal stromal tumours [42] or gene expression levels in testicular germ cell tumours [43].

In order to give physicians the opportunity to involve patients according to their wishes in the medical treatment process, attempts have repeatedly been made to predict patients' behaviour regarding participatory preferences by using sociodemographic and/or psychosocial factors [10,23,26,44]. However, only a single study by Adams and colleagues [26] has thus far been able to explain 48% of the variance in their model using a multiple linear regression analysis involving a group of Australian asthma patients. The present study seeks to predict the preferred decision-making roles of German patients with end-stage renal disease (ESRD) through the comprehensive application of sociodemographic and psychosocial factors. The employed factors consisted of age, educational level, information-seeking preference, trust in physician, and physicians' participatory decision-making (PDM) style. Using the specific method of prediction CFA, this study goes a step further with regards to a precise and robust means of identifying the types of patients wishing to participate in treatment decisions as well as those wishing to play a completely active or passive role as found in previous literature on this topic.

## Methods

### Sampling and data collection

The present study was conducted with patients of the non-profit Kuratorium fuer Dialyse und Nierentransplantation e. V. (KfH). 15000 of the 60992dialysis patients in Germany [45] are currently treated in the physician-run facilities of this institution. The KfH has implemented a quality assurance program QiN (Quality in Nephrology). All dialysis units of the KfH have the opportunity to take part in QiN voluntarily; in 2005 QiN was attended by 187 dialysis centres. QiN provides a large database of the included dialysis patients on various clinical data. In addition, QiN conducts a survey concerning health-related quality of life, once a year. Therefore, the 187 participating dialysis units obtained questionnaires from QiN for delivery to every patient who was treated in the unit during the fourth quarter of the year (October 1 to December 31, 2005). The staff of each unit was responsible for informing the patients about the purpose of the survey and the delivery of the questionnaire including a return envelope to the individual patient. The dialysis units were also responsible for sending the completed questionnaires back to QiN. The dialysis units received 14972 questionnaires for distribution among their patients. Since none of the individual dialysis centers kept count of how many survey questionnaires had actually been distributed to their patients, a concrete response rate could not be calculated. However, 6318 questionnaires were returned to QiN, which could be used for statistical analysis.

In the fourth quarter of 2005, questions concerning participation in treatment decision-making were included in the QiN health-related quality of life survey.

Whereas previous studies of people with renal disease focused on the decision between haemodialysis and peritoneal dialysis in the SDM process [17,46], this decision (haemodialysis) had already been made for the patients of this study. Nevertheless, there are various decisions that also need to be made during outpatient haemodialysis. For example, the patient can participate in the decision about how long, how often, at what time, and in which institution the dialysis should be administered, and how their comorbidities (e.g., hypertonia, diabetes) should be treated.

### Study design

**Figure 1:**
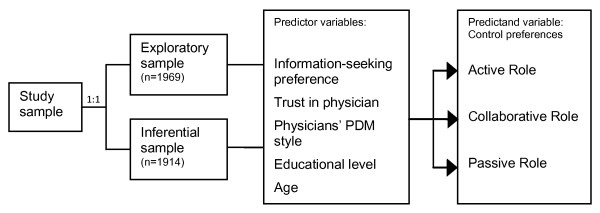
**Study design**.

For analyzing the present data, a modification of the configural frequency analysis was used, the so-called prediction configural frequency analysis. In conducting the prediction analysis, the overall sample of patients with no missing values was later randomly divided into two equal-sized subsamples [47]. In the first subsample, types were identified using the prediction CFA. In this process an attempt was made to predict the desired control preferences of the patients when making treatment decisions (active role, collaborative role, passive role) by using five predictor variables (information-seeking preference, trust in physician, physicians' participatory decision-making (PDM) style, educational level, and age). It is foreseeable that with many attributes the number of configurations and types, respectively, becomes so large that only sparse types can be detected. In this case one performs with one sample an exploratory CFA without α-adjustment. Then, within a second independent sample, inferential analyses are calculated only for those configurations which provided exploratory types in the first analysis. Therefore, in the second subsample, a prediction CFA was performed as cross-validation by way of the inferential verification (with α-adjustment [48]) of the types identified in the exploratory analysis (Figure 1).

### Configural frequency analysis

For a CFA, several attributes of a sample of subjects were observed. Each observed attribute consist though of different categories. Further, for each subject the value of each category is observed, which constitute the so called configurations. The number of individuals that a specific configuration exhibits is counted and these numbers are then recorded in a contingency table as absolute frequencies [34]. The values for each configuration

$${\text{X}}^{2}={\left(\text{observed frequency}–\text{expected frequency}\right)}^{2}/\text{expected frequency}$$

can be assessed under the null hypothesis of the total independence using critical values for the chi-square distribution for 1 degree of freedom [49].

In prediction CFA, one is interested in predicting certain configurations by means of other configurations. For this purpose, the attributes or variables are divided into two subsets: predictor variables and criterion variables. This means in the present study that information-seeking preference, trust in physician, physicians' participatory decision-making (PDM) style, educational level, and age are considered as predictor variables, while the control preferences scale is regarded as the criterion to be predicted. The absolute frequencies of all possible configurations are entered in a contingency table as in CFA. However, for each cell with a given frequency a fourfold table is derived. A prediction type is assumed if the probability of the joint incidence of the configurations is greater than under the null hypothesis on the independence of the variables. This is calculated by using Fisher exact tests [49]. A technical description and a calculation example for a (prediction) CFA is given in Additional file 1.

## Measures

### The control preferences scale (criterion variable)

The control preferences scale [10,50] identifies the type of participation patients wish to have in making treatment decisions. Patients have the opportunity to choose between five different statements about their desired type of participation. The options range from autonomous decision making by the patient (= active role; two response options), to participatory decision making between physician and patient (= collaborative role; one response option), to the physician having sole responsibility for decision making (= passive role; two response options). A "pick one approach" - a paper version of the scale that was originally developed as a card sorting approach - was used [8]. In the process, patients were asked to consider all five interaction styles and select the one that felt closest to their preferred role in treatment decision making.

### Predictor variables

The first predictor variable identified the patients' desire for information (Information subscale of the Autonomy Preference Index [22]). The scale consists of 8 items, which were answered on a Likert scale of 1 (strongly agree) to 5 (strongly disagree). Sum scores were calculated and two categories of the attribute (high and low) were established via median dichotomization.

The second predictor variable demonstrates the trust that the patients have in their physician [51]. The scale consists of 5 items, which were answered on a Likert scale with responses ranging from 1 (completely disagree) to 4 (completely agree). Since the median of the sum scores of this scale is also the maximum value, the mean was chosen for categorization, which was somewhat lower than the median. Thus, two prediction values are obtained: less than the mean value and greater than the mean value.

The third predictor variable corresponds to the patient's perceived involvement by the physician, measured by the Physicians' Participatory Decision-Making Style scale [52]. The scale consists of 3 items for which there are two different 5-point Likert response scales (e.g. "How often does your doctor ask you to take some of the responsibility for your treatment? (very often to not at all); If there were a choice between treatments, would your doctor ask you to help make the decision? (definitely yes to definitely no)"). Since the scale consists only of 3 items with very different response scales it was decided to categorize it by the individual item content rather than by calculating a sum score. However, because the Likert scale items are scaled in the same direction (positive - negative), it was resolved that at least two of the three scale items had to be answered in the same direction in order to identify two categories of the physician's PDM style (positive and negative).

The educational level and age of the patients constitute the fourth and fifth predictor variables. In the process, the educational level was subdivided into two categories (low: no secondary school certificate or lower secondary school certificate; high: secondary school certificate and higher, and age was subdivided into three categories (≤ 55 years; 56 - 75 years; ≥ 76 years). The categories for educational level and age were determined so that they fit the distribution of the data.

### Analyses

When analyzing the prediction configural frequency analysis, all configurations of the five predictors were generated. The five predictors were: M1 = Information-seeking preference (with 1 = low, 2 = high), M2 = Trust in physician (with 1 = < average, 2 = > average), M3 = Physicians' PDM style (with 1 = negative, 2 = positive), M4 = Educational level (with 1 = low, 2 = high) and M5 = Age (with 1 = ≤ 55 years, 2 = 56 - 75 years, 3 = ≥ 76 years). The predictors were supposed to predict the one-dimensional criterion M6 = Control preferences (with 1 = active role, 2 = collaborative role and 3 = passive role). As statistical verification of the configural types 2 × 2 × 2 × 2 × 3 × 3 = 144 Fisher exact tests were calculated. The prediction CFA was performed using the software developed by Krauth [53].

An α-adjustment (0.05) for multiple testing was performed by means of the Holm procedure, a more powerful approach than the Bonferroni procedure. Holm proposed a simple sequentially rejective procedure by ordering the p-values and comparing them with Bonferroni adjustments [48].

## Results

### Sample characteristics

In total, data were collected from n = 6318 haemodialysis' patients of the KfH via questionnaires. A comparison of the KfH patient population (about 15000 patients) to the "average German patient" with renal replacement therapy [45], which was only possible when comparing age and gender because of a lack of comparative data, showed that the KfH patients were representative of all dialysis patients in Germany regarding gender and age. In order to ensure that the overall study sample of 6318 patients was representative of the KfH patient population, both groups were compared with regard to their gender, age, educational level, length of their dialysis treatment, and their Karnofsky Index. The results showed the study sample to be representative of the overall population of KfH patients (data not shown).

Nonetheless, the patients of the exploratory and the confirmatory samples (n = 1969; n = 1914), with no missing values, are distinguishable from participants in the overall study sample (n = 6318) by a slightly lower average age (2 and 1 years less, respectively), a slightly higher educational level (3% more patients with a high educational level within the exploratory sample), a higher need for information (7% more patients with a high need for information within the exploratory and the confirmatory samples), and 3% more males within both subsamples. However, because of their minimal occurrence, these characteristics are not regarded as restrictive to the validity of the samples. No differences were found regarding the control preferences. As the patients were randomly assigned to either exploratory or confirmatory sample and as the two subsamples were large, it was expected that only differences due to random error would be found. The results were in line with this expectation (Table 1).

**Table 1:** Patient characteristics

	Exploratory sample (n = 1969)		Confirmatory sample (n = 1914)	
Gender				
Male	60.9%	(1199)	60.8%	(1164)
Female	39.1%	(770)	39.2%	(750)
Age (years)				
Mean (Range)	63.9	(18-94)	64.6	(20-96)
≤ 55 years	26.4%	(519)	24.1%	(462)
56 - 75 years	48.1%	(947)	48.9%	(935)
≥ 76 years	25.5%	(503)	27.0%	(517)
Years on dialysis				
Mean (Range)	4.6	(0-33)	4.8	(0-38)
Karnofsky performance index*				
Mean (Range)	74	(10-100)	74	(10-100)
Decision-making preference				
Active role	22.4%	(442)	22.2%	(424)
Collaborative role	31.3%	(616)	30.8%	(589)
Passive role	46.3%	(911)	47.1%	(901)
Educational level				
Low	65.9%	(1297)	68.6%	(1313)
High	34.1%	(672)	31.4%	(601)
Information-seeking preference				
Low	38.9%	(765)	38.9%	(744)
High	61.1%	(1204)	61.1%	(1170)
Trust in physician				
Below average	34.1%	(672)	35.4%	(677)
Above average	65.9%	(1297)	64.6%	(1237)
Physicians' PDM style				
Negative	38.1%	(750)	39.0%	(747)
Positive	61.9%	(1219)	61.0%	(1167)

* Karnofsky performance index: assessment tool used to assist clinicians/caretakers in measuring a patient's ability to carry out activities of daily living (e.g.: Normal, no complaints, no evidence of disease = 100; requires considerable assistance and frequent medical care = 50; Dead = 0).

### Prediction analysis

21 possible prediction types were identified (printed in bold) in the exploratory sample shown in Table 2. Consequently, the Fisher exact test of these configurations demonstrated a *p*-value < 0.05. Of the 21 possible prediction types that were identified, 8 configurations predicted the preference to play an active role in treatment decisions, 4 configurations predicted the desire to play a collaborative role, and 9 configurations predicted the desire to play a passive role. The 21 predictor configurations were then considered as hypotheses for the confirmatory prediction CFA.

**Table 2:** P-values of Fisher exact tests for the prediction configural frequency analyses of the exploratory and confirmatorysamples

Predictor configurations					Exploratory sample* (n = 1969)			Confirmatorysample^†^ (n = 1914)		
					M_6_			M_6_		
M_1_	M_2_	M_3_	M_4_	M_5_	1	2	3	1	2	3

1	1	1	1	1	0.868561	**0.013433**	0.965462	0.132716	0.250897	0.988444
1	1	1	1	2	0.986465	0.566994	0.065148	0.651998	0.589635	0.438021
1	1	1	1	3	0.837806	0.943782	**0.044112**	0.869598	0.816498	0.109900
1	1	1	2	1	**0.003995**	0.558877	0.998703	0.040347	0.743055	0.946179
1	1	1	2	2	0.206284	0.377986	0.945128	0.063365	0.643754	0.942566
1	1	1	2	3	0.167299	0.870319	0.760671	0.950949	0.766104	0.141515
1	1	2	1	1	0.387803	0.863616	0.492469	0.161224	0.974986	0.549439
1	1	2	1	2	0.736882	**0.012917**	0.979951	0.975624	0.845271	0.018431
1	1	2	1	3	0.999647	0.802734	**0.004485**	0.996089	0.357889	0.178549
1	1	2	2	1	**0.000526**	0.719929	1.000000	0.060949	0.046314	0.999981
1	1	2	2	2	0.428826	0.380616	0.855444	0.557708	0.357889	0.786593
1	1	2	2	3	1.000000	0.312849	1.000000	0.481830	0.924178	0.436117
1	2	1	1	1	0.857129	0.683738	0.290362	0.851363	0.441130	0.517129
1	2	1	1	2	0.999620	0.531546	**0.014243**	0.972254	0.923865	0.007423
1	2	1	1	3	0.967847	0.997609	**0.000459**	0.946666	0.936227	0.014773
1	2	1	2	1	0.404697	0.895093	0.584079	0.024306	0.890320	0.978174
1	2	1	2	2	0.835002	0.575279	0.370051	0.387784	0.052811	0.998311
1	2	1	2	3	0.869697	0.769805	0.284715	0.777927	0.269913	0.861121
1	2	2	1	1	0.490653	0.406689	0.752028	0.717236	0.823456	0.238753
1	2	2	1	2	0.980595	0.860401	**0.007893**	0.999999	0.718093	**0.000058 T1**
1	2	2	1	3	0.999730	0.946691	**0.000071**	0.999980	0.915213	**0.000060 T2**
1	2	2	2	1	0.122222	0.613263	0.908054	0.018876	0.421608	0.997229
1	2	2	2	2	0.506892	0.784709	0.393508	0.772922	0.589417	0.366223
1	2	2	2	3	0.978426	0.434567	0.216913	0.915578	0.889245	0.042856
2	1	1	1	1	**0.010568**	0.240021	0.999566	0.010179	0.650267	0.978838
2	1	1	1	2	0.120891	0.218840	0.981613	0.303068	0.058791	0.988485
2	1	1	1	3	0.771296	0.051227	0.952478	0.620639	0.103562	0.923111
2	1	1	2	1	**0.000000**	0.912684	0.999979	**0.000000 T3**	0.836925	0.999998
2	1	1	2	2	**0.000008**	0.897191	0.999555	0.010179	0.949048	0.869418
2	1	1	2	3	0.463636	0.093064	0.988886	0.319816	0.287885	0.970943
2	1	2	1	1	**0.013963**	0.206085	0.999954	0.029721	0.349747	0.995546
2	1	2	1	2	0.456875	**0.044917**	0.981564	0.079130	0.395506	0.968858
2	1	2	1	3	0.273647	0.928973	0.509020	0.996089	0.104609	0.473831
2	1	2	2	1	**0.000145**	0.411609	0.999990	0.007815	0.772473	0.980082
2	1	2	2	2	0.155768	0.233927	0.983029	0.099956	0.405697	0.971317
2	1	2	2	3	1.000000	0.068152	0.801432	0.077756	0.467936	0.993923
2	2	1	1	1	0.600728	0.713610	0.400621	0.034337	0.257264	0.997223
2	2	1	1	2	0.974082	0.757745	**0.031957**	0.996559	0.302793	0.092598
2	2	1	1	3	0.975795	0.121327	0.519333	0.855271	0.936875	0.036585
2	2	1	2	1	0.059371	0.977447	0.530169	0.114972	0.136279	0.997053
2	2	1	2	2	0.213526	0.769220	0.693151	0.087146	0.837020	0.789409
2	2	1	2	3	0.794185	**0.029025**	0.961348	0.961883	0.180994	0.682467
2	2	2	1	1	0.075093	0.803607	0.782896	0.106104	0.469835	0.936655
2	2	2	1	2	0.999969	0.467800	**0.001904**	0.999989	0.244953	0.006359
2	2	2	1	3	0.999868	0.745509	**0.001138**	0.991909	0.868168	0.004507
2	2	2	2	1	**0.001424**	0.431570	0.999327	**0.000080 T4**	0.312023	0.999994
2	2	2	2	2	0.750593	0.667267	0.246865	0.135057	0.574359	0.589893
2	2	2	2	3	0.490653	0.798727	0.380271	0.842434	0.711906	0.194625

Predictor variables: M_1 _= Information-seeking preference (1 = low, 2 = high), M_2 _= Trust in physician (1 = < average, 2 = > average), M_3 _= Physicians' PDM style (1 = negative, 2 = positive), M_4 _= Educational level (1 = low, 2 = high), and M_5 _= Age (1 = ≤ 55 years, 2 = 56-75 years, 3 = ≥ 76 years), and criterion variable: M_6 _= Control preferences (1 = active role, 2 = collaborative role, 3 = passive role). *possible prediction types are printed in bold. †confirmed prediction types are marked with a "T".

**Figure 2:**
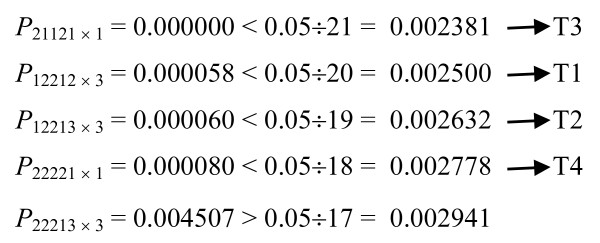
**α-Adjustment procedure**.

As verification of the prediction types, an alpha adjustment was performed, but only on the potential 21 types identified in the exploratory analysis (Figure 2). In the process, verification was provided for prediction type (12212)×3 labelled as "T 1", type (12213)×3 labelled as "T 2", type (21121)×1 labelled as "T 3" and type (22221)×1 labelled as "T 4".

It is predicted that dialysis patients preferring a passive role in treatment decisions (Type 1) demonstrate a low desire for information, have an above-average level of trust in their treating physician and have a positive perception of their physician's PDM style. In addition, these patients have a low educational level and are either between the ages of 56 and 75 or they are age 76 and older (Type 2).

Furthermore, it is predicted that patients with a high desire for information prefer to play an active role. In addition, these patients have a high educational level and are at most 55 years old. Furthermore, the trust that these patients have in their physicians can be characterized as below-average and they have a negative perception of their physicians' PDM style (Type 3). Type 4 patients show an above-average level of trust in their physician, and a positive perception of their physician's PDM style (Table 3).

**Table 3:** Confirmed Patient Types

	Passive Role	Passive Role	Active Role	Active Role
	Type 1 (n = 91)	Type 2 (n = 45)	Type 3 (n = 29)	Type 4 (n = 26)
Information-seeking preference	low	low	high	high
Trust in physician	> average	> average	< average	> average
Physicians' PDM style	positive	positive	negative	positive
Educational level	low	low	high	high
Age	56 -75 years	≥ 76 years	≤ 55 years	≤ 55 years

## Discussion

When predicting the control preference, the greatest contrast was found between the active and passive types' desire for information, their educational level and their age. In addition, the passive types always demonstrated above-average trust in their physician and a positive perception of their involvement by their physician in decision making (their physician's PDM style), whereas the active types could also exhibit a below-average level of trust in their physician together with a negative perception of their physician's PDM style (Table 3).

These results are supported in parts by the reports of other researchers: multivariate studies have already shown that the desire for active participation in treatment decision-making increases in patients with a higher educational level [26,44] and a somewhat lower age [44] and tends to decrease the older the patients and the lower their educational level [54]. These reports also found a correlation between a positive perception of the physician's involvement of the patient due to his PDM style and the desire for increased participation [26]. Kraetschmer and her colleagues [29] reported that patients preferring a passive role had a high level of trust in their treating physician and study participants preferring an active role had little trust in their physician. Hall et al. [55] found evidence for a generally high level of trust in the patients' physicians (90% or more of the patients). The trust values demonstrated by patients in this study could be explained in connection with the perceived values of the physician's PDM style: if there is a positive perception of the PDM style, then the patient's trust in the physician increases (Type 1, Type 2, Type 4). The opposite is also true: if there is a negative perception of the physician's PDM style, then the patient's trust in the physician decreases (Type 3). This study was not designed in such a way that causal direction between trust and perception of the physician's PDM style can be assumed. However, findings from literature suggest that trust of patients increases as a result of patients being satisfied with the physician's PDM style: although the development of a trusting relationship is not completely clarified, trust is believed to evolve over time and is shaped by patients' experiences and interactions with their physicians. This was shown on a sample of patients establishing a new physician-patient relationship: patients, who were involved in decisions as much as they wanted, expressed greater trust in their physicians after their first physician-patient contact [27].

Since "need for information" was the only attribute that was marginally higher in the exploratory and confirmatory subsamples (by 7%) than in the overall study sample of 6318 patients, particular attention was paid to predictor-criterion configurations with low p-values with regard to informational desire. If the predictor-criterion configurations (11121)×1 with *P*11121 × 1 = 0.040347 and (12221)×1 with *P*12221 × 1 = 0.018876 (Table 2, confirmatory sample) are therefore taken into account when interpreting the prediction types, the result is that the active role is preferred regardless of the informational desire of patients age 55 and younger with a high educational level. These patients have either a below-average level of trust in their physician together with a negative perception of the physician's PDM style or they have an above-average trust in their physician along with a positive perception of the physician's PDM style. Additionally, if the predictor-criterion configurations (22212)×3 with *P*22212 × 3 = 0.006359 and (22213)×3 with *P*22213 × 3 = 0.004507 (Table 2, confirmatory sample) are considered, the passive role is preferred regardless of the informational desire of patients with an above-average level of trust in their physician, a positive perception of their physician's PDM style, a low educational level and age older than 56 years (56 to 75 years old or older than 75). It was found that the informational desire of patients was independent of their preferred active or passive control preference. Although these results cannot be confirmed by cross-validation, they are supported by the literature on this topic: there have been several reports that although patients desired information about their upcoming treatment, they did not want to be involved in the treatment decisions [31,56]. Ende and his colleagues [22] also confirmed this in that they could find no correlation between the desire for information and the preferred decision-making roles of patients. The present study also showed that although the patients demonstrated a high desire for information, 47% of those surveyed wished to play a passive role in making treatment decisions.

Some of the associations that were revealed by the prediction configural frequency analysis could be supported by findings from the literature, as just presented. However, when two variables are associated only under the condition that one or more other variables take a specific value, Pearsons correlations would only be moderate and associations could not be revealed by the use of e.g. factor analysis. Though, their common occurrence would still be detected by means of CFA that is also sensitive when higher-order interactions between variables are present [37]. The prediction configural frequency analysis used to predict the control preferences of patients in making treatment decisions, took into account interactions between variables of the fifth order. Even though logistic regression and prediction CFA focus on the same data characteristics, on the relations among predictor variables and criterion, standard models of logistic regression (a variable-oriented approach) and prediction CFA (a person-oriented approach) vary. For this reason, results from both methods can not be compared or interpreted without further analyses and extension of the logistic regression model [57]. Nevertheless, it would be interesting to compare results between prediction CFA and other typal analyses or (logistic) regression models with regard to patients' preferences.

## Limitations

When interpreting the findings of this study, consideration needs to be given to a number of methodological issues. The participating patients were recruited at 187 dialysis centres. Thus the design of this study was in fact a multilevel approach, which was not considered in the analyses. Therefore, the results might be biased, if interactions between physicians' behaviour and dialysis centres may occur. However, since the number of participating dialysis units was very high (187), this was not considered as threatening to the validity of the study results.

In order to ensure that the overall study sample was representative of the KfH patient population, it was shown that both groups were comparable with regard to their gender, age, educational level, length of their dialysis treatment, and their Karnofsky Index. However, it might be that the relevant motives to participate in this study or not are psychological of nature, which may introduce selection bias.

The prediction CFA was performed as cross-validation by splitting the original sample into two subsamples. However, it might be interesting to confirm the study results in further independent populations.

Due to the selected study group of chronically ill patients with end-stage renal disease, the results of this study may only be carefully transferred to other physician-patient interactions or as well as other patient groups. As a follow-up study, it would still be interesting to verify whether the prediction types found in this study using the above-mentioned procedure can be confirmed in other populations such as e.g. breast cancer patients.

The generalisability of results may further be limited by errors associated with survey non-response and item non response.

In addition, the current condition of the chronically ill patients could differ widely from one another: some of the patients might have decided on the treatment of their comorbidities and some patients on changing their health behaviour. Although the seriousness of such a decision and its implications could affect the decision-making preferences, it is not clear to what extent this would influence the results of the present study. Future studies on medical decision-making would benefit from including some measures of the patients medical condition. Additionally, some patients may have had difficulties in understanding the role descriptions of the control preferences scale [58], whose effects on the results cannot be assessed.

Furthermore, the questionnaire was given at a single time in the course of treatment while trust is a factor that is assumed to vary with time and according to the clinical experiences and outcomes. However, the assessment of trust in our study is based on long-term experiences of patients. All patients that took part in the survey have been under long-term treatment in the participating facilities. Most of them were able to establish a stable relationship with their physician over time which is based more or less on trust depending on the character of the relationship.

Ethical approval for this survey was obtained by the Ethical Committee Board of the University Hospital of Cologne.

## Conclusions

The prediction CFA approach is new to the field of patient participation and could show in contrast to the above mentioned research studies, how a particular control preference role is determined by an association of five variables. Further research should aim at increasing the percentage of participants representing a type by carefully choosing different combinations of psychological, social and medical parameters. The more accurate patient types become, the better will be the ability of the treating physicians to meet the specific needs of each patient concerning participation in treatment decisions. This is of particular importance when establishing a long-term relationship with chronically ill patients.

## Competing interests

The authors declare that they have no competing interests.

## Authors' contributions

SL was responsible for the study design, data analysis, interpretation of results, and drafting the manuscript. OO contributed to interpretation of results, drafting and revising the manuscript. CK and FS contributed to study design and interpretation of results. AW and CB were responsible for data gathering and contributed to the design of the study. HP contributed to the study design, drafting and revising the manuscript. All authors read and approved the final manuscript.

## Pre-publication history

The pre-publication history for this paper can be accessed here:

http://www.biomedcentral.com/1472-6947/10/47/prepub

## Supplementary Material

Additional file 1 **Calculation of a prediction CFA**. The method prediction configural frequency analysis is described in detail and demonstrated by calculating an example. Click here for file
