# Supplementary material for: Configural frequency analysis as a method of determining patients' preferred decision-making roles in dialysis
Source: BMC Med Inform Decis Mak. 2010 Sep 11;10:47. doi: 10.1186/1472-6947-10-47 (PMC3161344; doi:10.1186/1472-6947-10-47)
Supplement: Additional file 1 — Calculation of a prediction CFA. The method prediction configural frequency analysis is described in detail and demonstrated by calculating an example. [file 1472-6947-10-47-S1.pdf]

Additional file 1

Configural frequency analysis

The starting point for the analysis is a sample of n subjects, whose t attributes M1,…,Mt were observed. Each of these attributes has r different categories (e.g., attribute M1 has r1 categories, attribute M2 has r2 categories, etc.). Overall, there are r1 x r2 x r3 x … x rt different configurations. The number of individuals that a specific configuration exhibits is counted and these numbers are then recorded in a t-dimensional contingency table as absolute frequencies [34]. However, it is possible that some configurations occur with a high probability, simply because the individual categories of the configurations have a high probability. To prevent a false identification of types due to a random combination of certain (predictor) variables, a chance correction is implemented. Using the chance correction, the expected value for a configuration is calculated under the null hypothesis of total independence of the variables and then subtracted from the absolute (observed) frequency of the configuration. Because the probabilities of the occurrence of the attribute categories are generally unknown, one acts on the assumption of a conditional probability of the configuration for fixed one-dimensional marginal frequencies and can then calculate the conditional expected values through simple multiplication. The values for each configuration

X^2 = (observed frequency – expected frequency)^2 / expected frequency

can be assessed under the null hypothesis of the total independence using critical values for the chi-square distribution for 1 degree of freedom [49].

In prediction CFA, one is interested in predicting certain configurations by means of other configurations. For this purpose, the t attributes or variables M1,…,Mt are divided into two subsets: one part of the t variables that can be considered as predictor variables, is denoted as s variables. The remaining variables (t-s) are

supposed to be criterion variables. This means in the present study that of all 6 variables s=5 are considered as predictor variables, while the remaining variable (t-s=6-5=1) is regarded as the criterion to be predicted. The absolute frequencies of all possible configurations (s x (t-s)) are entered in a contingency table as in CFA. However, for each cell with a frequency f(s x (t-s)) a fourfold table is derived by considering in addition f(s) - f(s x (t-s)), f(t-s) - f(s x (t-s)), and n - f(s) - f(t-s) + f(s x (t-s)). A prediction type is assumed if the probability of the joint incidence of the configurations s x (t-s) is greater than the conditional probability for given marginal frequencies under the null hypothesis on the independence of s and (t-s). This is calculated by using Fisher exact tests [49].

Calculation of a prediction CFA

As an example, the prediction CFA of the predictor-criterion configuration (12212)×3 (T 1) is demonstrated as follows: There have been 91 patients expressing this particular configuration (Table 4). The corresponding fourfold table is shown in Table 5.

Table 5: Fourfold table for predictor-criterion configuration (12212)×3

f(s x (t-s)) 91	*f(s) - f(s x (t-s))* 54	*f(s)* 145
f(t-s) - f(s x (t-s)) 810	*n - f(s) - f(t-s) + f(s x (t-s))* 959	*n - f(s)* 1769
f(t-s) 901	*n - f(t-s)* 1013	*n* 1914

Is only a pocket calculator available, one uses the normal approximation of Fisher's exact test. The z-value is being calculated [53]:

$$Z_{(s \bullet (t-s))} = \frac{\left(f\,(s \bullet (t-s)) - \frac{f\,(s) \bullet f\,(t-s)}{n} - 0.5\right) \bullet n \bullet \sqrt{n-1}}{\sqrt{f\,(s) \bullet (n - f\,(s)) \bullet f\,(t-s) \bullet (n - f\,(t-s))}}$$

Using the fourfold table entries, this yields to:

$$Z_{12212x3} = \frac{\left(91 - \frac{145 \cdot 901}{1914} - 0.5\right) \cdot 1914 \cdot \sqrt{1913}}{\sqrt{145 \cdot (1914 - 145) \cdot 901 \cdot 1013}} = 3.848279$$

The corresponding P-value is:

$$P_{12212x3} = 1 - \Phi(3.848) = 1 - 0.999940 = 0.000059$$

This value can be found in table 2 (T1 = 0.000058) with a slight difference due to rounding errors. Since this value is smaller than the adjusted $\alpha^* = 0.05 \div 20 = 0.0025$ one assumes a prediction type (T1).

Table 4: Frequencies for the prediction configural frequency analyses of the confirmatorysample

Predictor configurations					Confirmatory sample† (n = 1914)			
					M_6			
M_1	M_2	M_3	M_4	M_5	1	2	3	Σ
1	1	1	1	1	7	8	5	20
1	1	1	1	2	11	16	26	53
1	1	1	1	3	5	8	19	32
1	1	1	2	1	8	5	6	19
1	1	1	2	2	9	7	8	24
1	1	1	2	3	1	3	8	12
1	1	2	1	1	4	1	5	10
1	1	2	1	2	7	14	34	55
1	1	2	1	3	1	8	13	22
1	1	2	2	1	7	9	1	17
1	1	2	2	2	5	8	9	22
1	1	2	2	3	2	1	4	7
1	2	1	1	1	2	5	7	14
1	2	1	1	2	8	14	38	60
1	2	1	1	3	5	8	25	38
1	2	1	2	1	4	1	1	6
1	2	1	2	2	3	6	1	10
1	2	1	2	3	1	3	2	6
1	2	2	1	1	4	5	12	21
1	2	2	1	2	12	42	91 T1	145
1	2	2	1	3	3	15	45 T2	63
1	2	2	2	1	9	7	4	20
1	2	2	2	2	6	10	17	33
1	2	2	2	3	5	8	22	35
2	1	1	1	1	13	9	9	31
2	1	1	1	2	17	27	23	67
2	1	1	1	3	7	14	12	33
2	1	1	2	1	29 T3	13	9	51
2	1	1	2	2	13	6	12	31
2	1	1	2	3	3	4	2	9
2	1	2	1	1	11	10	7	28
2	1	2	1	2	16	17	18	51
2	1	2	1	3	1	10	11	22
2	1	2	2	1	12	7	8	27
2	1	2	2	2	9	9	8	26
2	1	2	2	3	4	3	1	8
2	2	1	1	1	12	12	8	32
2	2	1	1	2	11	31	50	92
2	2	1	1	3	7	9	26	42
2	2	1	2	1	8	10	5	23
2	2	1	2	2	10	7	12	29
2	2	1	2	3	1	6	6	13
2	2	2	1	1	16	17	20	53
2	2	2	1	2	29	78	130	237
2	2	2	1	3	12	24	56	92
2	2	2	2	1	26 T4	20	12	58
2	2	2	2	2	22	24	33	79
2	2	2	2	3	6	10	20	36
		Σ			424	589	901	1914

Predictor variables: M_1=Information-seeking preference (1=low, 2=high), M_2=Trust in physician (1= <average, 2= >average), M_3=Physicians' PDM style (1=negative, 2=positive), M_4=Educational level (1=low, 2=high), and M_5=Age (1= ≤55years, 2=56-75years, 3= ≥76years), and criterion variable: M_6= Control preferences (1=active role, 2=collaborative role, 3=passive role). †confirmed prediction types are marked with a "T".
